# A Comment on “Deep Proteogenomics of a Photosynthetic Cyanobacterium”

**DOI:** 10.1021/acs.jproteome.5c00796

**Published:** 2026-05-21

**Authors:** Haijun Liu, Himadri B. Pakrasi, Michael L. Gross

**Affiliations:** † Department of Biology, 7547 Saint Louis University, St. Louis, Missouri 63103, United States; ‡ Department of Biology, Washington University in St. Louis, St. Louis, Missouri 63130, United States; § Department of Chemistry, Washington University in St. Louis, St. Louis, Missouri 63130, United States

**Keywords:** proteogenomic, cyanobacteria, proteome annotation, liquid chromatography tandem mass spectrometry (LC-MS/MS), false discovery

## Abstract

Proteomic researchers strive to achieve complete annotation of protein-coding DNA sequences to provide a foundational context for their relevant biological data. A recent deep proteogenomic study using a photosynthetic cyanobacterium *Synechocystis sp*. PCC 6803 by Spät et al. proposed 64 refined open reading frames (ORFs). By searching LC-MS/MS data from affinity chromatography-isolated protein complexes, our laboratory identified that six of these high-abundance ORFs possess N-terminal initiation start sites that differ than those proposed in the alternative models. Our findings are supported by highly confident MS2 data, phylogenetic analysis, chemical labeling, and established data from two independent research groups. Based on these high-quality experimental identifications, we subsequently propose a standardized strategy and set of criteria for future deep proteogenomic efforts to ensure accurate and stringent proteogenomic annotation.

## Graphical abstract

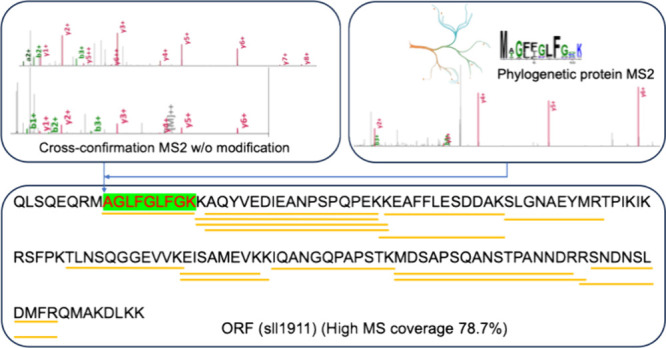

## Introduction

Proteogenomics integrates high-throughput liquid chromatography-tandem mass spectrometry (LC-MS/MS) with genomic data to refine the genome annotations. This methodology enhances our understanding of gene expression and noncanonical translation products, such as small open reading frames (ORFs). However, the field faces challenges such as the vast dynamic range of protein abundances, where high-abundance proteins mask critical regulatory molecules during data collection. Despite improving analytical capacities, bottlenecks remain that lead to false-positive identifications due to chemical diversity and incorrect databases.^1,2^ Establishing a strategic workflow is essential to prevent overannotation as data volumes grow.

N-Terminomics is a critical subfield focused on characterizing a proteome’s N-termini to understand protein maturation and stability. Its objectives include annotating translational start sites and identifying modifications such as N-terminal acetylation. Yet, many current reannotations lack solid experimental support, causing false discoveries through less stringent searches and a lack of standardized pipelines.

A recent study by Spät et al. (2023) showcased deep proteogenomics in the photosynthetic cyanobacterium *Synechocystis sp*. PCC 6803.^3^ Analyzing over 18 million MS/MS spectra using their data and data in PRIDE,^4^ the authors claimed to refine 64 ORFs, including eight novel annotations. For instance, they presented ORF Sll1911 as a refined model based on the identification of an additional upstream peptide, QLSQEQR. This proposal directly contradicts current UniProt^5^ (P73253, or Sll1911) annotations and our own mass spectrometry evidence. In this comment, we present high-quality MS2 data and proposal-specific criteria for future proteogenomic efforts. By leveraging purified protein complexes rather than whole-cell lysates, we identified six ORFs that demonstrate the importance of stringent experimental annotation. While this biochemical approach requires more effort and offers lower throughput, it provides the accuracy necessary for reliable genome refinement.

## Material and Methods

### Synechocystis sp. PCC 6803 Strains for Protein Complex Isolation

*Synechocystis sp*. PCC 6803 (*Synechocystis* 6803 hereafter) C-terminally His_6_-tagged strains were grown in BG11 medium at 30 °C and a light intensity of 30 μmol of photons m^–2^ s^–1^ with supplementary corresponding antibiotics. C-terminally his_6_-tagged OCP (OEOCP, OCP overexpression cell line) was reported in reference.^6^ C-terminally his_6_-tagged FRP (OEFRP, FRP overexpression cell line) was a gift from Prof. Kirilovsky’s group.^7^ C-terminally His_6_-tagged CP47 (His47) was constructed as reported.^8^ *ΔctpA* and *Δpsb27* were provided by the Pakrasi group.^9^

### Sample Preparation and Mass Spectrometry Analysis

*In vivo* protein cross-linking/modification using OEOCP and OEFRP cells was performed by using membrane permeable cross-linker DSS with minor modifications.^10,11^ Briefly, dithiobis­[succinimidylpropionate] DSS dissolved in dimethyl sulfoxide (DMSO) was added to the resuspended cells (0.5 mM Chlorophyll *a*) in HEPES buffer (50 mM, pH 8.0) to give a final concentration of 20 mM. The cell/DSS mixture was incubated for 1 h at room temperature with gentle shaking. A final concentration of 50 mM Tris was used to quench the reaction. Protein complex isolation in OEOCP and OEFRP cells was reported in references, respectively.^6,7^ Isolation of Photosystem II (or PSII), a pigment–protein complex involved in photochemical water splitting in photosynthesis, followed previous published protocols^8,12^ with modifications.^9^ Trypsin digestion and LC-MS/MS analysis followed reference.^13^ Briefly, protein pellets were dissolved in an 8 M urea solution (20 μL) followed by incubation with tris­(2-carboxyethyl)­phosphine (2.5 mM) at 37 °C for 30 min and treated with iodoacetamide (5 mM) for 30 min at room temperature. After LysC digestion (0.05 μg/μL) for 2 h followed by 8× dilution, the protein solution was further incubated with trypsin overnight at 37 °C. Finally, the digestion was quenched by adding 0.1% of formic acid.

Genetically modified (mutant) and unmodified protein complexes were prepared from *Synechocystis* 6803, and then submitted to *in vitro* cross-linking analysis. BS_3_ was incubated with PSII samples for ∼30 min in the dark at 25 °C with 10-, 50-, 100-fold excess of cross-linker. Cross-linked protein solution was desalted and purified by acetone precipitation before enzymatic digestion. The peptide mixture was analyzed on a Q Exactive Plus mass spectrometer (ThermoFisher Scientific) coupled with the Ultimate 3000 reversed-phase HPLC system. For detailed LC-MS/MS acquisition, please refer to our previous methodological development report.^11^ Peptide and cross-linked peptides were identified using pFind^14^ and pLink,^15^ respectively. Weblogo of protein sequence was constructed by following the literature.^16^

### Processing and Database Searching of LC-MS/MS Data

pFind (version 3.2.2) and pLink (version 3.0.16) software studio was used for peptide and cross-links identification and analysis, with settings as precursor tolerance ± 10 ppm, fragment tolerance ± 20 ppm, enzyme specificity (full), missed cleavages (up to 3), fixed modifications (carbamidomethyl [C]), variable modification (first round, open search; second round, Xlink_ DSS[138]­[K], DSS[156]­[K]). In the result filter section in the software pFind, FDR ≤ 1%, Protein search database was used in the literature.^3^ Protein sequence data (.fasta) were from literature^3^ or annotation of each concerned six ORFs annotated UniProt^5^ (http://uniprot.org).

### Validation of Refined ORFs

All MS2 spectra were manually checked by focusing on the peak assignments of y and b ions, fragmented ion coverage/intensity, isotopic feature (monoisotopic, first isotope), neutral loss, *a*-ion shifting, etc. Mass shifts resulting from either natural or artificial (instrumental) modifications were confirmed referring to the database^17^ (https://web.expasy.org/findmod/findmod_masses.html, e.g., acetylation (42.01)).

## Results and Discussion

In our research, the purified or enriched protein complexes were first subjected to LC-MS/MS analysis and then to manual identification, focusing on the quality of the precursor and product-ion spectra. Here, we provide the mass spectrometry data supporting that 6 (out of 64) ORFs have specified translation initiation sites different than the recently proposed refinement,^3^ i.e., 5 of them uphold the established annotations in Uniprot database;^5^ 1 has a translation site that is different than either the refinement in the literature^3^ or the annotation in Uniprot^5^ (Table 1), making it a novel ORF. In this work, we present our findings on the N-terminoproteogenomic refinements of the genes/proteins of interest, leaving their biological significance (e.g., Sll1911) for a separate report. We are motivated to share this short communication because an effective strategy for N-terminoproteogenomics remains missing in the current literature/community. All 6 ORFs (plus one ORF tll0889 from another organism, Table 1) are identified in high abundance in our analysis.

**Table 1:**
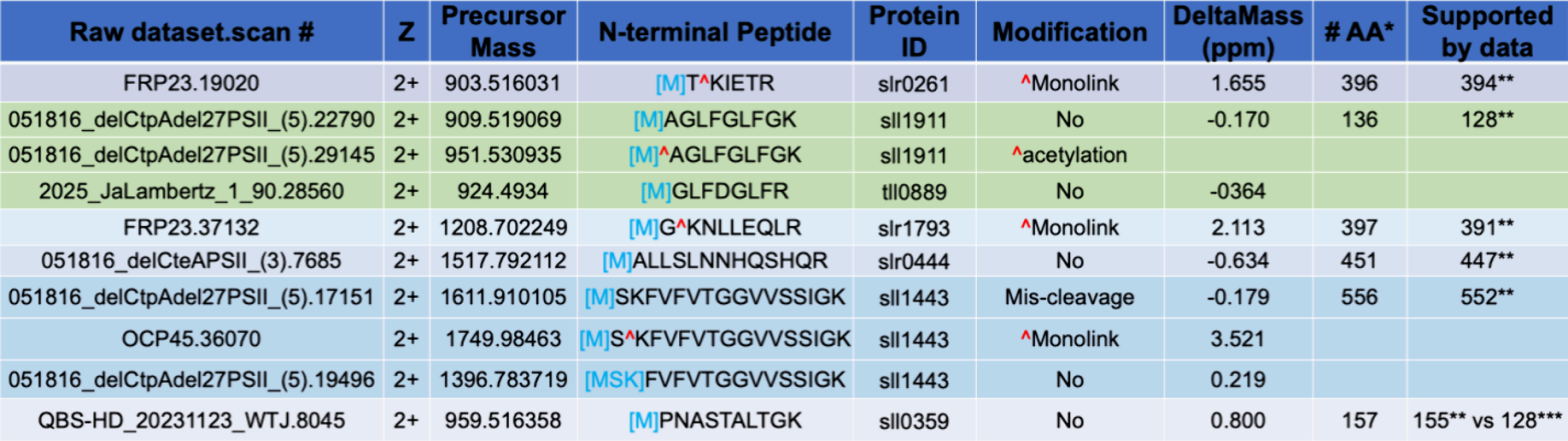
MS/MS Identification of N-Terminal Peptide

The translation initiation codon is the codon on an mRNA that is placed on the P site of a ribosome small subunit to begin protein synthesis. This codon specifies the first amino acid in the polypeptide elongation. AUG is generally considered as the standard initiation codon, which encodes methionine (Met, or M) in both eukaryotes and prokaryotes (modified formyl-methionine (fMet) in prokaryotes). Additionally, prokaryotic genes have long been known to initiate at non-AUG start codons, ∼12% GUG (Val, or V) and ∼8% UUG (Leu, or L) and variable incidence of AUU (Ile) and AUC (Ile).^18,19^ The initiator amino acid (iMet, ifMet) is usually removed via a post-translational modification that occurs after protein synthesis starts, a process called methionine excision (NME) catalyzed by methionine aminopeptidases. Whether the iMet/ifMet is removed or not depends primarily on the second amino acid, also called P1′ residue immediately following the methionine. iMet/ifMet is removed if the P1′ residue has a small, uncharged side chain, such as Ala, Cys, Gly, Pro, Ser, Thr, or Val. It is retained if the P1′ residue is bulky or charged, such as Asp, Glu, His, Ile, Leu, Lys, Met, Asn, Phe, Gln, Arg, Trp, Tyr). NME affects protein stability, localization, and N-terminal modifications (e.g., acetylation to give a mass shift 42.01); the modification has roles in protein–protein interactions and degradation. Identification of N-terminus of a protein consequently plays an important role in defining its basic biological function.

N-Terminal acetylation (Nt-acetylation) is a common, irreversible post-translational modification (PTM) that occurs on the α-amino group of the first amino acid of a protein after removal (or retention) of the initiator methionine. Nt-acetylation can be partial for some proteins, meaning not all copies of a protein are acetylated at the N-terminus. This is clearly observed in Sll1911 (Table 1, Figure 1). In our database searching, using the protein sequence suggested by reference,^3^ we failed to identify the peptide QLSQEQR, which was reported as a peptide in Sll1911 ^3^ and Figure 1d of the literature.^3^ We have confidently identified a peptide AGLFGLFGK, indicating that it is the most N-terminal peptide sequence of ORF Sll1911 (Figure 1A,B). Here, we can also conclude that it is the removal of the ifMet that results in the observed protein sequence in the mature protein (Figure 1B). Additionally, the P1′ residue (Ala in this case) is partially acetylated (note Figure 1B vs [Fig fig1]C). These data strongly support that Sll1911 indeed starts with an Met. In the previous report a peptide QLSQEQR was identified^3^ (sequence also shown in Figure 1A before the Met). Presently, no reports showed that proteins’ translation initiation can take place with a Gln (Q). If Gln, as an exception, is the start codon of Sll1911, it is highly possible that a peptide of SMAG­LFGL­FGK should be identified which is preceded by a R, a trypsin cleavage site (Figure 1A,D), rather than peptide AGLFGLFGK and its acetylated form (Figure 1B,C). Furthermore, Tll0889, a homologous protein of Sll1911, from *Thermosynechococcus vestitus* BP-1, a bacterium, is identified in a novel heterodimeric PSII complex by an independent group^20^ (PXD033676). The most N-terminal peptide of Tll0889 indicates that the initiating Met is removed, leaving peptide GLFDGLFR as the most N-terminal peptide (Figure 1E,F). Please note that although the peptide sequence upstream of Met in both Sll1911 and Tll0889 do not share sequence homology (Figure 1A vs [Fig fig1]E), it is observed that there is high sequence homology of the most N-terminal peptide of Sll1911 and Tll0889 (Figure 1G). In this case, mass spectrometry data sets from a different organism can lend ideas and provide strong cross-confirmational evidence based on so-called phylogenetic perspective (Figure 1G).

**Figure 1:**
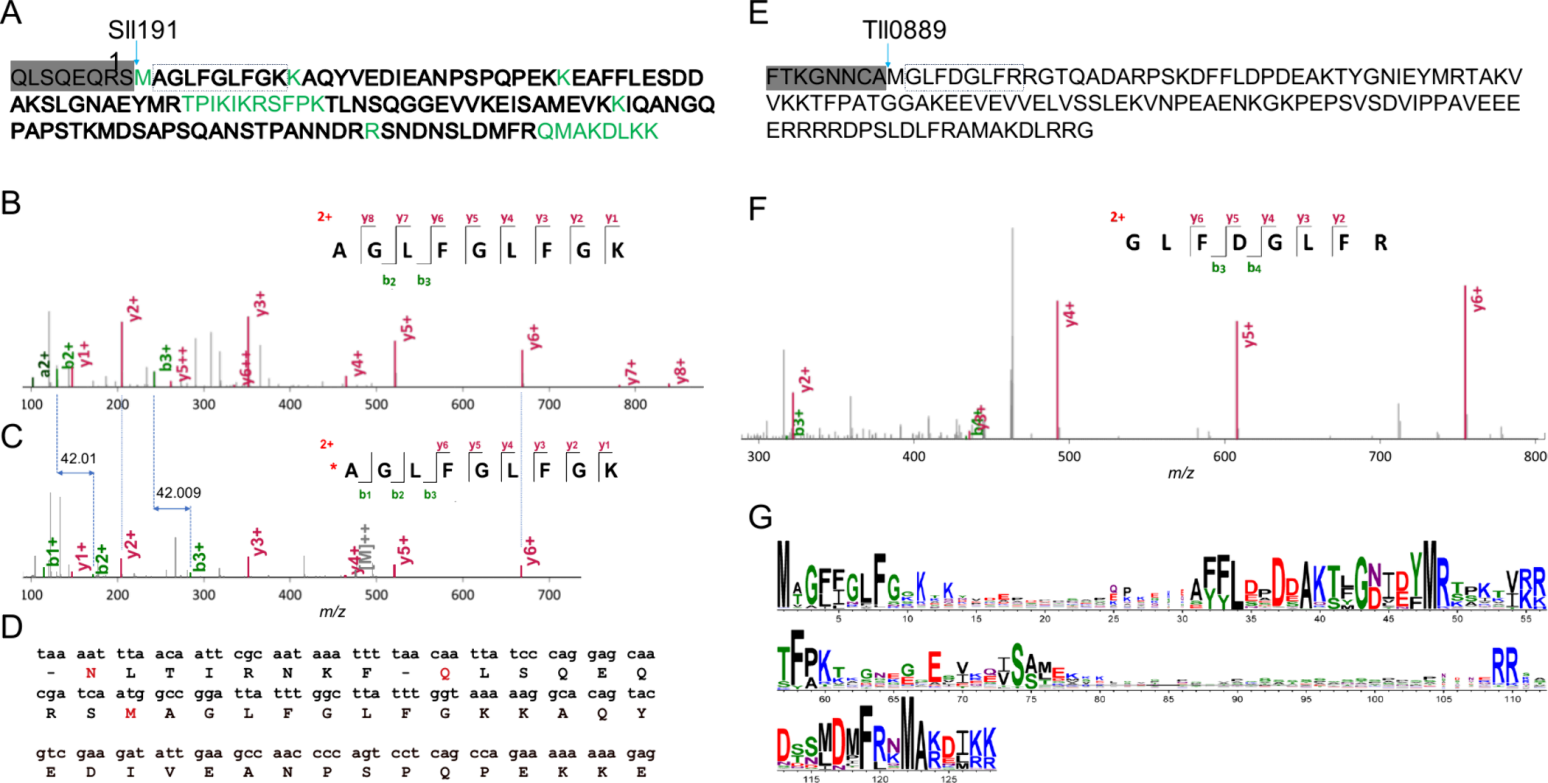
A. Protein sequence of Sll1911, including its upstream peptide QLSQEWRS (shaded) which was claimed to be identified by Spät et al. in literature. Amino acid sequences in green are not identified in the prior work. B. MS2 spectra of Sll1911 with initial fMet posttranslationally removed, and C. MS2 spectra of the acetylated peptide form of B (on N-terminal A) with indicated mass (*m*/*z*) shift of 42.01, characteristic of acetylation modification. D. Genomic and proteomic context of translation initation of Sll1911. E. *tll0889*, a homologue protein sequence of Sll1911, in *Thermosynechococcus vestitus BP-1*. F. MS2 spectra of the most N-terminal peptide of Tll0889 by Lambertz et al., PXD033676, no b ions were detected below 300 (*m*/*z*). G. Sequence logo of Sll1911 family protein, generated by using https://weblogo.threeplusone.com/create.cgi by using Sll1911 homologue protein sequences (from ∼100 organisms).

We believe that identification of the most N-terminal peptide of ORFs with high confident MS2 spectra is the key to differentiate the translation start codon. The high coverage of y and b ions of Slr0444 (92.3% and 46%, respectively) and those of Sll0359 (90% and 60% respectively) support that the correct translation initiation site should be Met. It should be noted that, Figure 2B (the most N-terminal peptide of Slr0444 starting with MALL···) support the gene annotation of Slr0444 in Uniprot^5^ (https://www.uniprot.org/uniprotkb/Q59975/entry), but not the one given by Spät et al.,^3^ (dark FVPSMALL···Figure 2A). Interestingly, identification of PNASTALTGK (Figure[Fig fig2]D, Sll0359) does not support either the claim by Spät et al.,^3^ (157 aa) or the Slr0359′s annotation in Uniprot^5^ (P74426) (155 aa vs 128, Table 1), indicative of a refined novel ORF. Additionally, peptide PNASTALTGK of Sll0359 was identified in both our data sets and a publicly available data set in PRIDE (PXD050522) deposited by the Qiu’s group.^21^ In this case, refinement of ORF Slr0359 is supported not only by high confident MS2 data in our hands but also by the data from an independent colleague laboratory.

**Figure 2:**
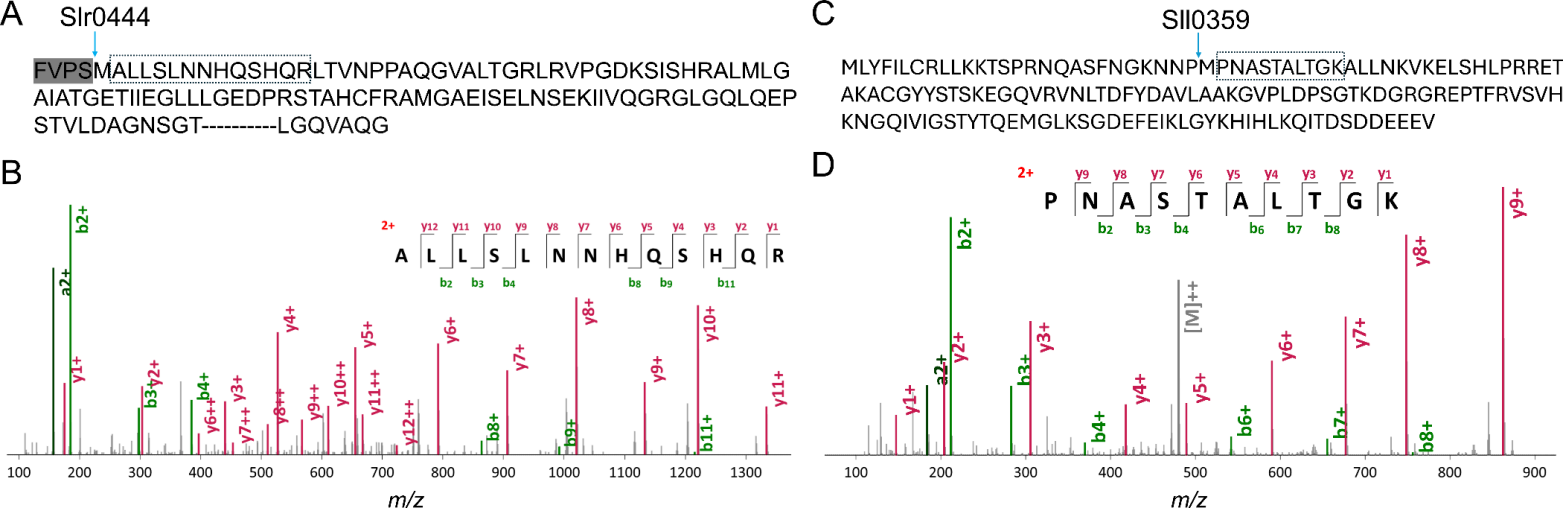
A and B, protein sequence of ORF Slr0444 (upstream of Met, partial protein sequence) and its most N-terminal peptide MS2 spectra, respectively. C and D, protein sequence of Sll0359 (upstream of Met included) and its most N-terminal peptide MS2 spectra, respectively.

Identifying a short and most N-terminal peptide of a protein using mass spectrometry generally poses a challenge. This happens when a trypsin cleavage site is located too near the translation initiation methionine. Short peptides (1–3 amino acids, Figure 3A, Sll1443, MSK, or SK if M is removed) may not ionize well in MS or poorly retainted in LC or may be lost during sample preparation. All these factors make the detection of short peptides by MS/MS very difficult or even not possible. However, there are strategies of improving the identifications of the likely most N-terminal peptide, such as the use of alternative protease, or chemical labeling of N-terminal peptides (followed by enrichment), blocking trypsin cleavage site, top-down proteomics, or semitryptic digestion. In our effort, we have used (hydrophobic, membrane permeable) DSS ((Disuccinimidy)­suberate), a homobifunctional amine-reactive cross-linker that reacts with primary amines, mainly the ε-amino group of lysine residues or the N-terminal α-amino group of proteins. Cross-linking modification of Lysine with DSS (or BS3, bis­(sulfosuccinimidyl)­suberate) can subsequently block the action of trypsin cleavage (a mis-cleavage), resulting in a longer peptide that is to be likely identified. It is a very useful approach to preserve the most N-terminal peptides. Figure 3B shows the most abundant peptide FVFV­TGGVV­SSIGK resulting from a trypsin cleavage site that is close to the N-terminus, leaving a dipeptide SK (following Met) unidentified. Due to our sample was initially subjected to DSS modification, we are now able to identify a peptide S^KFVF­VTGG­VVSS­IGK with the K (Lys) monolink modified by the used cross-linker in our reaction. Monolink modification apparently abolishes the trypsin cleavage site. Additionally, Figure 3C shows a MS2 spectrum of the peptide SKFV­FVTG­GVVS­SIGK resulting from partial digestion with one miscleavage. Briefly, all b ions on the C-terminal side of the modified peptide S^KFV­FVTG­GVVS­SIGK have an increased mass of 138.069 Da than the corresponding b ions in the mis-cleavage peptide SKFV­FVTG­GVVS­SIGK, with identical y ions (two reference y ion, y2 and y13, Figure 3C,D). Figure 4 shows other two more most N-terminal peptide of protein Slr1793 and Slr0261 using this strategy, but these two protein was annotated as having alternative start in the literature^3^ and our data support the correct annotation in UniProt^5^ (Slr1793 and Slr0261 in UniProt.org).

**Figure 3:**
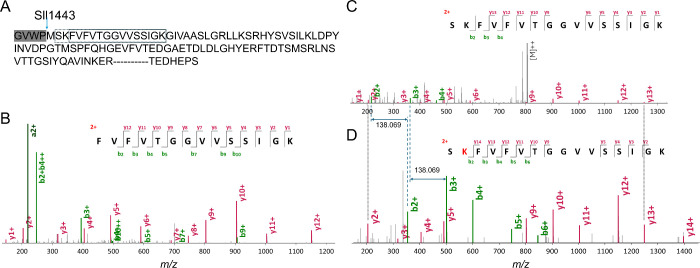
A. Protein sequence of ORF Sll1443 (upstream of Met, partial protein sequence) and (B). its truncated N-terminal peptide MS2 spectra, (C) the most N-terminal peptide (with one mis-cleavage via partial digestion, and (D) most N-terminal peptide with K modified by DSS which eliminated the trypsin digestion (mis-cleavage via chemical modification), respectively.

**Figure 4:**
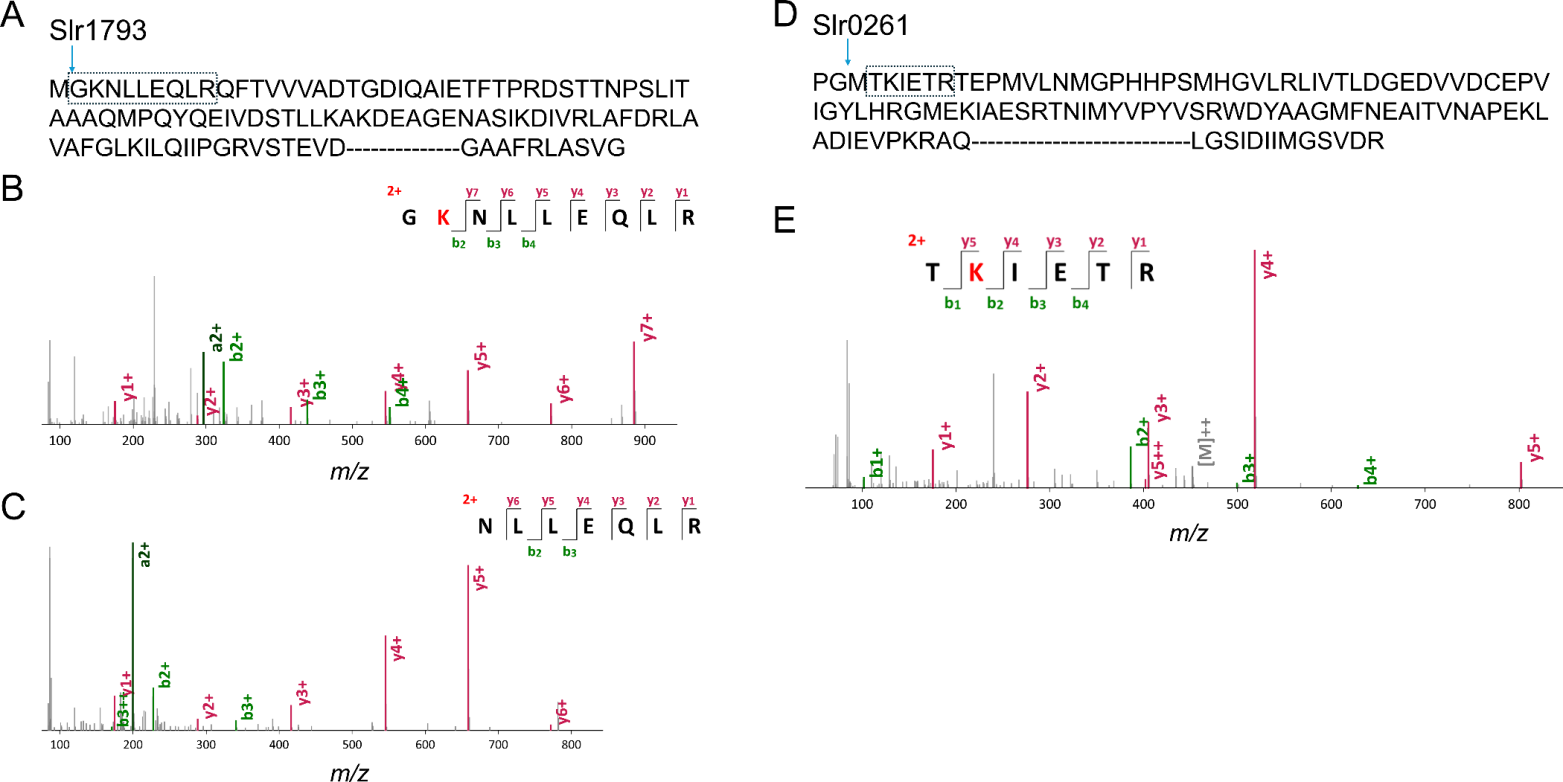
A, B, C, Protein sequence of ORF Slr1793, MS2 spectra with K modified by DSS and MS2spectra without modification at K (2 position). D, E, protein sequence of ORF Slr0261 and MS2 spectra with K modified by DSS which eliminated the trypsin digestion, respectively.

## Conclusions

In our analysis, the N-terminal peptides for all six ORFs were confidently identified, as demonstrated by the high-quality MS2 data presented in Figures 1–4. To enhance the rigor of future research and prevent overannotation, we propose a practical strategy and a set of criteria for deep proteogenomic initiatives and protein N-terminomics:

1. **Mandatory Spectral Evidence:** All claimed novel ORFs must be supported by high protein sequence coverage from LC-MS/MS analysis. Comprehensive MS2 spectra should be presented in the main text or provided as a Supporting Information PDF if more than five novel ORFs are identified.
2. **Optimized Proteolysis**: If a protease cleavage site is situated too close to the potential initiation methionine—preventing the detection of short peptides—researchers should utilize partial digestion or alternative proteases to effectively capture the N-terminal fragment.
3. **Chemical blocking**: chemical labeling of protease sites, such as modifying lysine residues with NHS-ester reagents like DSS or BS3, should be employed to block specific cleavage points. This preserves longer, identifiable N-terminal peptides that would otherwise be lost.
4. **Phylogenetic validation**: phylogenetic analysis of related proteins in different organisms should be performed whenever corresponding LC-MS/MS data are available to support the proposed initiation methionine.
5. **Targeted enrichment**: whenever possible, the protein or protein complex of interest should be isolated using genetic tagging followed by affinity chromatography. While this biochemical approach is time-consuming, the resulting improvement in sequence coverage and spectral quality adds significant confidence to the annotation.

We maintain that the accurate annotation of every protein justifies these extensive efforts as it is foundational to our understanding of biological structure and function.

## Data Availability

All data needed to evaluate the conclusions in the paper are present in the paper. Two previously deposited databases in PRIDE (Proteomics IDEntifications) were used for this communication^20,21^ with PRIDE IDs PXD033676 and PXD050522, respectively.
